# Effect of Endometrial Injury on Secretion of Endometrial Cytokines and IVF Outcomes in Women with Unexplained Subfertility

**DOI:** 10.1155/2015/757184

**Published:** 2015-10-26

**Authors:** Yu Liang, Junyan Han, Chanwei Jia, Yanmin Ma, Yonglian Lan, Ying Li, Shuyu Wang

**Affiliations:** ^1^Assisted Reproduction Center of Beijing Obstetrics and Gynecology Hospital, Capital Medical University, Beijing 100026, China; ^2^Institute of Infectious Diseases, Beijing Ditan Hospital, Capital Medical University, Beijing 100011, China; ^3^Beijing Key Laboratory of Emerging Infectious Diseases, Beijing 100011, China

## Abstract

In order to determine the effect of endometrial injury (EI) on *in vitro* fertilization (IVF) outcomes in women with unexplained subfertility and explore the relationship between EI and endometrial inflammatory cytokines, 66 women with unexplained subfertility undergoing IVF treatment were recruited. 38 patients in the EI group underwent EI in the mid-luteal phase of the cycle and 28 patients in the non-EI (NEI) group. According to the pregnancy outcome, the NEI and EI groups were divided into NEI-nonpregnant (NEI-NP), NEI-pregnant (NEI-P), EI-NP, and EI-P. All patients underwent aspiration of endometrial secretions immediately before embryo transfer. The concentrations of ten mediators were measured using Milliplex Magnetic Bead assay. The clinical pregnancy was significantly higher in the EI than in the NEI group. The concentrations of interleukin- (IL-) 6, IL-8, IL-12 (p70), IL-13, interferon- (IFN-) *γ*, monocyte chemotactic protein- (MCP-) 1, and vascular endothelial growth factor (VEGF) were significantly higher in the EI than the NEI group. The expression of IFN-*γ* and VEGF in the EI-P was significantly increased compared to the EI-NP group. These findings suggest that, in women with unexplained subfertility, endometrial injury might be a potential method to improve clinical pregnancy rates by promoting the expression of IFN-*γ* and VEGF.

## 1. Introduction

Successful implantation is dependent on the development of high-quality embryos and the acquisition of endometrial receptivity. Although the fertilization rate is relatively high and embryo culture conditions continue to improve, implantation is still the limiting step in the success of* in vitro* fertilization-embryo transfer (IVF-ET) [1]. In humans, the uterus becomes receptive during the mid-secretory phase (days 19–23) of the menstrual cycle, which is known as the window of implantation (WOI). Implantation is a process of embryonic attachment to the endometrium and subsequent invasion into the stroma of the uterine wall. Implantation is a complicated and multiple process involving various cytokines and growth factors, along with a dialogue between the embryo and the endometrium [2].

Various factors have been proven to contribute to the success of implantation [3]. Several studies have suggested a favorable effect of endometrial injury (EI) on the implantation success rate, especially in women with recurrent implantation failure [4–9], while other small trials failed to detect any benefit [10, 11]. With respect to the mechanism of injury-induced improvement of endometrial receptivity, Kalma et al. reported that local injury to the endometrium causes significant changes in the pattern of expression of genes related to implantation [12]. Gnainsky et al. suggested that injury-induced local injury induces an inflammatory reaction which favors implantation [13]. Natural killer cells, macrophages, and dendritic cells are recruited to the injured site and increased quantities of cytokines, growth factors, and chemokines are secreted, thus resulting in successful implantation [13]. Both studies were conducted during days 20–23 of spontaneous menstrual cycles [13, 14]. During menstruation, the endometrium is shed and controlled ovarian hyperstimulation (COH) can cause significant changes in endometrial secretory profiles [14].

Compared with spontaneous menstrual cycles preceding IVF, endometrial receptivity at the time of ET has been shown to be more important and directly related to ET outcomes [15]; however, the effect of endometrial injury on cytokine expression and endometrial receptivity at the endometrial-embryo interface has not been determined. It has previously been shown that it is possible to analyze the markers of receptivity through endometrial secretions obtained immediately prior to ET without disrupting embryo implantation [16]. In the current study we collected endometrial secretions immediately before ET, detected the concentration of inflammatory cytokines, chemokines, and growth factors, and explored the effect of endometrial injury on cytokine profiles and IVF outcomes.

## 2. Materials and Methods

### 2.1. Patients

This prospective study was conducted between March 2013 and October 2014 in the Assisted Reproduction Center of Beijing Obstetrics and Gynecology Hospital, Capital Medical University. Ethical approval was received from the local institutional Ethics Committee and written informed consent was obtained from all participants. Women attending the center with indications for IVF treatment were recruited. The patients in the endometrial injury (EI) group were selected based on their desire to participate, while the nonendometrial injury (NEI) group consisted of patients in contemporaneous IVF cycles who declined endometrial injury.

The inclusion criteria were as follows: women with unexplained subfertility with an indication for IVF treatment underwent first IVF cycle; unexplained infertility is a term used to describe infertile couples in whom standard investigations, including semen analysis, tests of ovulation, and tubal patency, have failed to detect any gross abnormality; it is a diagnosis of exclusion [17]; a normal uterine cavity demonstrated by hysteroscopy within 6 months; <35 years of age; body mass index (BMI) >19 or <30 kg/m^2^; basal follicle-stimulating hormone (bFSH) <12 mIU/mL; and regular menstrual cycles. Subjects were excluded from recruitment due to the presence of ovarian hyperstimulation syndrome, endometrial thickness <8 mm, and having no good quality embryos for transfer. The quality of partners' semen of recruited women was normal.

Sixty-nine women with unexplained infertility were initially recruited for the study. The patients were divided into two groups: EI group (*n* = 39) and NEI group (*n* = 30). One patient in the EI group was excluded due to the presence of ovarian hyperstimulation syndrome. Two patients in the NEI group were excluded because of endometrial thickness <8 mm (*n* = 1) and having no good quality embryos for transfer (*n* = 1). Therefore, there were 38 women in EI group and 28 women in NEI group. There was no significant difference in the baseline characteristics between the two groups (Table 1).

### 2.2. Endometrial Injury

All patients underwent transvaginal ultrasound in the follicular phase of the spontaneous menstrual cycle preceding the IVF treatment cycle to monitor follicle growth and determine follicular rupture. EIs were performed 5–7 days after ovulation. The procedure was performed in a standard fashion using a Pipelle catheter (Pipelle de Cornier, Laboratoire C.C.D., Paris, France). The Pipelle catheter was passed through the cervix up to the uterine fundus. The piston was drawn back to the end of the sheath to create a negative pressure. The sheath was rotated 360 degrees and moved back and forth between the fundus and internal os at least three to four times during a period of 30 s before it was gently withdrawn to ensure covering the entire endometrium and endometrial tissue had been obtained [11]. All procedures were performed by the same surgeon, who was experienced in gynecological operations.

### 2.3. Ovarian Stimulation and IVF

All patients started their IVF treatment in the subsequent cycle with ovarian stimulation using the mid-luteal long protocol, as previously described [18]. GnRH agonist (Triptorelin, Ferring, Germany) was initiated at 0.1 mg daily dose on the 21st day of the preceding spontaneous cycle. On days 2-3 of the menstrual cycle, the patient underwent a transvaginal ultrasound examination and the serum estradiol level was measured. Once a suitable degree of downregulation had been achieved, human menopausal gonadotrophin (hMG; Menogon, Ferring GmbH, Kiel, Germany) or recombinant FSH (Puregon; Organon, Dublin, Ireland or Gonal F; Merck Serono S.p.A., Modugno, Italy) was administered with a starting dose of 150–300 IU per day based on the antral follicle count, age, and body mass index (BMI), according to standard operating procedures. Ovarian response was monitored by serial transvaginal scanning and hormonal monitoring. Drug dosage was further adjusted based on the ovarian response. When one to three leading follicles were ≥18 mm in diameter, 250 *μ*g of Ovidrel (Merck Serono S.p.A.) was administered to trigger final maturation of the oocytes. Oocyte retrievals were performed 36 h later. Two embryos were transferred 2-3 days later. Excess good quality embryos were frozen for subsequent transfer.

### 2.4. Aspiration of Endometrial Secretions

Aspiration of endometrial secretions was performed immediately before ET. While in the lithotomy position, the patient's cervix was exposed and cleansed after insertion of the speculum. A 2 mL syringe was connected to an ET catheter (Wallace, SIMS Portex Ltd., Hythe, Kent, UK). The external catheter was inserted through the cervix with the inner catheter tip shielded within the external catheter to avoid contamination with cervical mucus. Once within the uterine cavity, the inner catheter is advanced, and suction is applied for 30 s before the inner catheter is again withdrawn within the external catheter, which is withdrawn from the uterus [19]. Because the aspirates are highly viscous, aspirate volumes could not be reliably measured but were between 1 and 4 *μ*L. The tip of the catheter is cut off and snap frozen in liquid nitrogen in an Eppendorf tube and stored at −80°C [20].

### 2.5. Detection of Cytokine Profiles in Endometrial Secretions

Assay buffer was added to recover the samples from the frozen tip catheter and the samples were diluted to 100 *μ*L with Assay Buffer for analysis of cytokines. Ten soluble cytokines, chemokines, and growth factors (vascular endothelial growth factor [VEGF]) in aspirates of endometrial secretions were detected by multiplex analysis using the Milliplex Magnetic Bead assay (Millipore, Billerica, MA, USA). The proinflammatory cytokines include interleukin- (IL-) 1*β*, IL-12 (p70), interferon- (IFN-) *γ*, and tumor necrosis factor- (TNF-) *α*. IL-13 has more anti-inflammatory properties, and IL-6 has both pro- and anti-inflammatory characteristics. Three chemokines were included in the assay (IL-8, eotaxin, and monocyte chemotactic protein- (MCP-) 1). The Luminex 200TM system and Milliplex Analyst were used for detection and analysis.

### 2.6. Outcome Measures

The primary outcome was the clinical pregnancy rate. The secondary outcomes were the concentrations of cytokines and implantation rate. The implantation rate was the number of sacs detected on ultrasound divided by the number of embryos transferred. Clinical pregnancy was defined as the presence of at least one gestational sac on ultrasound at 6 weeks.

### 2.7. Statistics

Statistical comparisons were carried out using the Mann-Whitney *U* test, chi-square test, and Student's *t*-test, where appropriate, with the Statistical Program for Social Sciences (SPSS; version 18.0). A two-sided *P* < 0.05 was considered statistically significant.

## 3. Results

### 3.1. Impact of Endometrial Injury on IVF Outcomes

Univariate analysis comparing treatment characteristics between the EI and NEI groups is provided in Table 2. There was no significant difference in the baseline characteristics between the two groups; however, the clinical pregnancy rates were significantly higher in the EI group compared to the NEI group (63.2% versus 35.7%, *P* = 0.04). The differences in implantation rate (38.2% versus 21.4%, *P* = 0.05) approached statistical significance but were not statistically significant. These results suggested that EI significantly improved IVF outcomes.

### 3.2. Impact of Endometrial Injury on the Cytokine Profile

**Figure 1:**
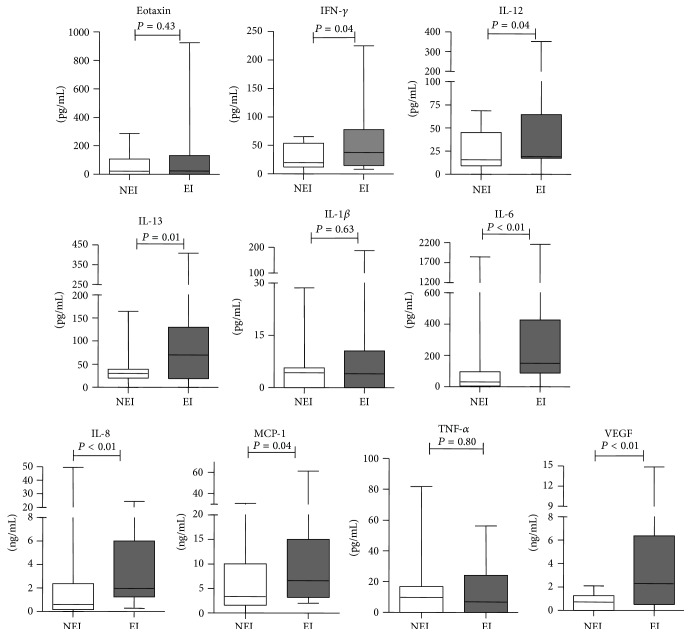
Concentrations of soluble mediators in aspirates of endometrial secretions in the EI and NEI groups. The expression of cytokine profiles [eotaxin, IFN-*γ*, IL-1*β*, IL-6, IL-8, IL-12 (p70), IL-13, TNF-*α*, MCP-1, and VEGF] of endometrial secretions obtained before ET from 66 patients (NEI: 28; EI: 38) was analyzed. The box plot horizontal lines represented the median and the 25th–75th percentile. The *P* values obtained are the results of the Mann-Whitney *U* test.

To explore whether or not EI influenced the cytokine profile of endometrial secretions, we determined the cytokine profiles of endometrial secretions obtained before ET from 66 patients (NEI, *n* = 28; EI, *n* = 38) using the Milliplex Magnetic Bead assay. The concentrations of IFN-*γ*, IL-6, IL-8, IL-12 (p70), IL-13, MCP-1, and VEGF in endometrial secretions in the EI group were significantly higher than the NEI group (Figure 1). Thus, EI might induce a local inflammatory response by stimulating the expression of inflammatory cytokines, chemokines, and growth factors.

### 3.3. Impact of Inflammatory Cytokines on IVF Outcomes

**Figure 2:**
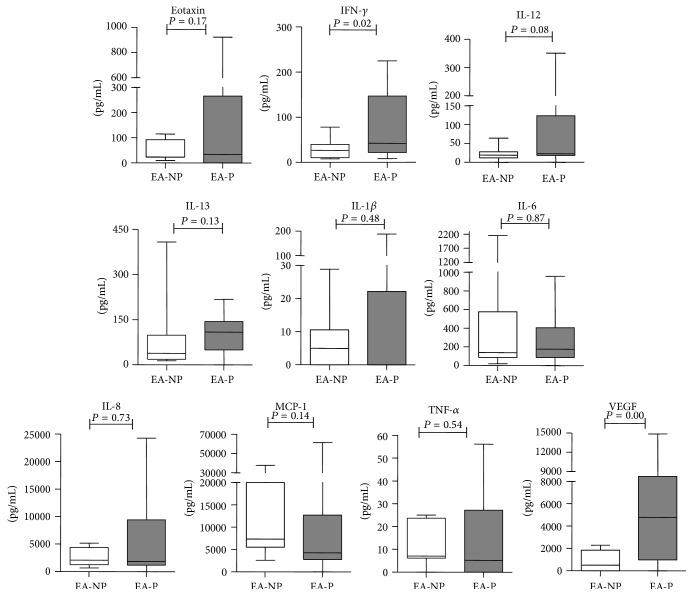
Concentrations of soluble mediators in aspirates of endometrial secretions in the EI-NP and EI-P groups. The expression of cytokine profiles [eotaxin, IFN-*γ*, IL-1*β*, IL-6, IL-8, IL-12 (p70), IL-13, TNF-*α*, MCP-1, and VEGF] in endometrial secretions obtained before ET from 38 patients (EI-NP: 14; EI-P: 24) was analyzed. The box plot horizontal lines represented the median and the 25th–75th percentiles. The *P* values obtained are the results of the Mann-Whitney *U* test.

**Figure 3:**
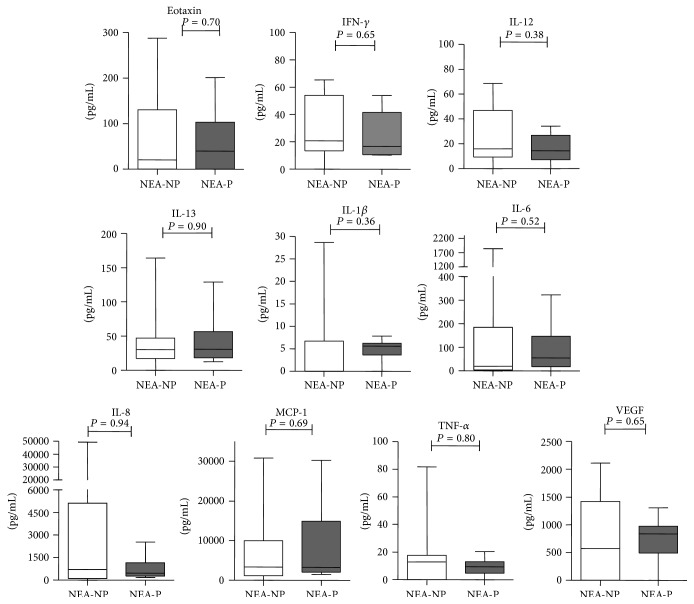
Concentrations of soluble mediators in aspirates of endometrial secretions in the NEI-NP and NEI-P groups. The expression of cytokine profiles [eotaxin, IFN-*γ*, IL-1*β*, IL-6, IL-8, IL-12 (p70), IL-13, TNF-*α*, MCP-1, and VEGF] in endometrial secretions obtained before ET from 28 patients (NEA-NP: 18; NEA-P: 10) was analyzed. The box plot horizontal lines represented the median and the 25th–75th percentiles. The *P* values obtained are the results of the Mann-Whitney *U* test.

To further study the effect of injury-induced inflammation on uterine receptivity and outcome, according to the pregnancy outcome after IVF treatment, the NEI and EI groups were divided into two subgroups, respectively, NEI-nonpregnant (NEI-NP), NEI-pregnant (NEI-P), EI-nonpregnant (EI-NP), and EI-pregnant (EI-P). The expressions of cytokines, chemokines, and growth factors were explored in the aspirates of endometrial secretions of the above four groups. Compared with the EI-NP group, the concentrations of IFN-*γ* and VEGF in aspirates of endometrial secretions of the EI-P group were significantly increased (Figure 2). While there was no significant difference in all of ten cytokines in aspirates of endometrial secretions between NEI-NP and NEI-P group (Figure 3). Thus, EI might promote the secretion of IFN-*γ* and VEGF and further impact the IVF outcomes in women with unexplained subfertility.

## 4. Discussion

The current study demonstrated that EI in the cycle preceding ovarian stimulation significantly improved the clinical pregnancy rate of IVF-ET in women with unexplained subfertility. In addition, our results suggested that EI might promote the expression of IFN-*γ* and VEGF and further improve the pregnancy outcome. Our results supplemented the mechanism by which EI impacted pregnancy outcome at the time of ET.

The role of EI on IVF was controversial. Barash et al. [4] first demonstrated that EI during the spontaneous cycle preceding IVF treatment doubled the rates of implantation, clinical pregnancy, and live births in women with repeated implantation failures. Then, several studies confirmed the positive effect of EI on embryo implantation and clinical pregnancies at different time and with different frequencies [5–7]; however, conflicting results were reported. Karimzade et al. [10] evaluated the effect of local injury to the endometrium on the day of oocyte retrieval on implantation and pregnancy rates in women undergoing the first IVF cycle, and the results demonstrated that local injury to the endometrium disrupted the receptive endometrium and had a negative impact on implantation and IVF outcomes. Yeung et al. [11] demonstrated that EI induced by endometrial aspiration in the luteal phase of the preceding cycle does not improve the ongoing pregnancy rate in unselected subfertile women undergoing IVF. Thus, the population, timing, technique, and frequencies of EI were variable and led to different outcomes.

Among the previous studies, Barash et al. focused on the women with repeated implantation failures and performed endometrial biopsies on days 8, 12, 21, and 26 of the cycle prior to treatment, which resulted in more than twofold increase in clinical pregnancy and live birth rate per ET compared to control patients [4]. In contrast with Barash et al., we recruited the women with unexplained infertility as subjects. There is limited data reporting the use of endometrial injury in unexplained infertility undergoing their first IVF treatment. In addition, the patients in our study underwent EI once, 5–7 days after ovulation of the spontaneous menstrual cycle preceding the IVF treatment cycle. It had proved that performing EI only during the secretory phase was sufficient to ameliorate the outcome of IVF [21]. It could effectively relieve the discomfort of patients.

In the current study, we showed that EI had a positive effect on the clinical pregnancy rates. Gibreel et al. performed EI in the luteal phase of the spontaneous menstrual cycle in 54 women with unexplained subfertility and advised them to practice sexual intercourse according to their convenience for the next 6 months [17]. Then they found significantly higher clinical pregnancy rates among the patients with EIs compared to the control group (*n* = 51) [17]. In agreement with the study of Gibreel et al., women with unexplained subfertility were recruited and the results were consistent with previous findings [17]. Because an impairment of endometrial receptivity may be the etiology for couples diagnosed with unexplained subfertility [22–24], we suggest that EI may facilitate the preparation of a receptive endometrium in women with unexplained subfertility. More studies are needed to confirm the effect of EI on IVF in women with unexplained subfertility.

The mechanism underlying EI action has not been elucidated. One study showed that the success of implantation was secondary to the development of an inflammatory reaction induced by trauma [25]. Gnainsky et al. [26] demonstrated that EI upregulated the expression of proinflammatory cytokines that recruited monocyte/macrophages to the site of injury, which in turn triggered the stromal and epithelial cells to express some particular implantation-associated genes and are involved in the apposition and adhesion of the blastocyst. To exclude the interference of inflammatory cytokines by other factors, such as hydrosalpinx and endometriosis, women with unexplained subfertility were recruited. In the current study, the analysis of cytokines in the endometrial secretion revealed a significant increase in the expression of IFN-*γ*, IL-6, IL-8, IL-12, IL-13, MCP-1, and VEGF in the patients who underwent EI in the cycle preceding IVF treatment. This result supported the previous hypothesis and implied that the injury-induced inflammatory response might affect the following IVF cycle. This long-term effect may rely on the fact that monocytes recruited to injured sites are long-lived and reside in some tissues for months [27]. Furthermore, monocytes can differentiate into tissue-resident macrophages/dendritic cells in response to cytokines that are expressed during the WOI [27].

To further study the effect of injury-induced inflammation on uterine receptivity and outcome, we compare the concentrations of ten cytokines between NEI-NP and NEI-P groups and EI-NP and EI-P groups, respectively. Boomsma et al. analyzed 17 soluble regulators of implantation in endometrial secretions aspirated from 210 women undergoing IVF and showed that individual mediators had no significant associations with pregnancy. In their study, the patients were unselected infertile women and did not undergo EI. In our study, all of the concentration of ten cytokines had no difference between NEI-NP and NEI-P groups, which were consistent with Boomsma et al. However, compared with the EI-NP group, the expression of IFN-*γ* and VEGF in aspirates of endometrial secretion in the EI-P group was significantly increased.

IFN-*γ* facilitates uterine vascular modification, decidualization, and uterine natural killer cell differentiation in mice [28]. In humans, IFN-*γ* has been identified as an essential regulatory pathway benefiting vascular modeling [29, 30] and first trimester extravillous cytotrophoblast migration [31]. Recent findings indicated that IFN-*γ*-secreting NK cells play a proangiogenic role and promote VEGF expression [32, 33]. VEGF is a major modulator of vascular growth and remodeling required for the cyclic blood vessel proliferation in the endometrium [34–36] and improves endometrial and subendometrial blood flow. The localization of VEGF could be identified in both glandular and luminal epithelial cells and most cells of stroma in human endometrium at all stages of the menstrual cycle [37]. EI might stimulate epithelial cells and stromal cells secrete VEGF. In addition, EI could recruit neutrophils and macrophages into endometrium [38], and these recruited neutrophils and macrophages could produce VEGF and IFN-*γ* [39, 40].

We therefore hypothesized that EI might facilitate the expression of IFN-*γ* and VEGF, thus providing a favorable environment promoting vascularization in maternal decidua during early pregnancy. In our study, VEGF, the regulator of angiogenesis, was upregulated after EI, which might explain the previous observations that EI could increase the endometrial blood flow perfusion [41]. In addition, patients with unexplained infertility have higher impedance blood flow in spiral arteries compared with fertile controls, which means that impaired blood flow could be an important contributing factor to unexplained infertility [42]. Hannan et al. revealed that VEGF levels are significantly reduced in uterine fluid during the midsecretory phase in women with unexplained infertility compared with fertile women [43]. And they support the concept that endometrial secretions, including VEGF, play important roles during implantation [43]. Thus, in women with unexplained subfertility, EI might be a potential way to improve endometrial receptivity and IVF outcomes.

Taken together, EI can in some content regulate the level of cytokines, chemokines, and growth factors in endometrial secretions immediately before ET. Clinically, EI might be a potential method to improve clinical pregnancy rates in women with unexplained subfertility by promoting the expression of IFN-*γ* and VEGF. The major limitation of the study is its nonrandomised nature, raising the possibility of bias. However we have shown that the two treatment allocations did not differ significantly in any clinical or IVF parameter which reduces the probability that significant bias may be influencing results. Because of the limited sample size in our study, the effect of EI on the clinical pregnant rate and cytokine profiles needs further study.

## Figures and Tables

**Table 1:** Clinical parameters of the NEI and EI group parameter.

	NEI (*n* = 28)	EI (*n* = 38)	*P*
Age (year)	29.85 ± 3.12	30.22 ± 2.51	0.60
BMI (kg/m^2^)	22.10 ± 3.38	21.75 ± 2.50	0.63
bFSH (IU/L)	6.42 ± 1.57	6.72 ± 1.60	0.44
bE_2_ (ng/mL)	37.49 ± 10.93	32.80 ± 13.80	0.14
bLH (IU/L)	4.12 ± 1.43	3.87 ± 1.62	0.53
Antral follicles	11.25 ± 3.01	11.15 ± 4.73	0.93

Clinical parameters of the EI (*n* = 38) and NEI (*n* = 28) group. BMI: body mass index; bFSH: basal follicle-stimulating hormone; bE_2_: basal estradiol; bLH: basal luteinizing hormone.

**Table 2:** Treatment characteristics of the NEI and EI group.

Parameter	NEI (*n* = 28)	EI (*n* = 38)	*P*
Total Gn dose (U)	2803.61 ± 846.20	2783.62 ± 852.59	0.93
E_2_ on HCG day (ng/mL)	3797.20 ± 1465.89	3150.03 ± 1341.87	0.07
Retrieved oocytes	11.96 ± 3.99	10.08 ± 4.35	0.08
Endometrial thickness (mm)	10.91 ± 1.85	10.71 ± 1.70	0.65
Implantation rate (%)	21.4	38.2	0.05
Clinical pregnancy rate (%)	35.7	63.2	0.04^*∗*^

Treatment characteristics of the EI (*n* = 38) and NEI (*n* = 28) group. Gn: gonadotropins; E_2_: estradiol; HCG: human chorionic gonadotropin.

^*∗*^Statistically significant (*P* < 0.05).
